# Identification and Functional Analysis of Pheromone and Receptor Genes in the *B3* Mating Locus of *Pleurotus eryngii*

**DOI:** 10.1371/journal.pone.0104693

**Published:** 2014-08-18

**Authors:** Kyung-Hee Kim, Young Min Kang, Chak Han Im, Asjad Ali, Sun Young Kim, Hee-Jeong Je, Min-Keun Kim, Hyun Su Rho, Hyun Sook Lee, Won-Sik Kong, Jae-San Ryu

**Affiliations:** 1 Environment-friendly Research Division, Gyeongsangnam-do Agricultural Research and Extension Services, Jinju, Republic of Korea; 2 Herbal Medicine Research Division, Korea Institute of Oriental Medicine (KIOM), Daejeon, Republic of Korea; 3 Department of Microbiology, Gyeongsang National University, Jinju, Republic of Korea; 4 Mushroom Research Division, National Institute of Horticultural and Herbal Science, Rural Development Administration, Eumsung, Republic of Korea; Georg-August-University of Göttingen Institute of Microbiology & Genetics, Germany

## Abstract

*Pleurotus eryngii* has recently become a major cultivated mushroom; it uses tetrapolar heterothallism as a part of its reproductive process. Sexual development progresses only when the *A* and *B* mating types are compatible. Such mating incompatibility occasionally limits the efficiency of breeding programs in which crossing within loci-shared strains or backcrossing strategies are employed. Therefore, understanding the mating system in edible mushroom fungi will help provide a short cut in the development of new strains. We isolated and identified pheromone and receptor genes in the *B3* locus of *P. eryngii* and performed a functional analysis of the genes in the mating process by transformation. A genomic DNA library was constructed to map the entire mating-type locus. The *B3* locus was found to contain four pheromone precursor genes and four receptor genes. Remarkably, receptor PESTE3.3.1 has just 34 amino acid residues in its C-terminal cytoplasmic region; therefore, it seems likely to be a receptor-like gene. Real-time quantitative RT-PCR (real-time qRT-PCR) revealed that most pheromone and receptor genes showed significantly higher expression in monokaryotic cells than dikaryotic cells. The pheromone genes *PEphb3.1* and *PEphb3.3* and the receptor gene *PESTE3.3.1* were transformed into P5 (*A3B4*). The transformants were mated with a tester strain (*A4B4*), and the progeny showed clamp connections and a normal fruiting body, which indicates the proposed role of these genes in mating and fruiting processes. This result also confirms that *PESTE3.3.1* is a receptor gene. In this study, we identified pheromone and receptor genes in the *B3* locus of *P. eryngii* and found that some of those genes appear to play a role in the mating and fruiting processes. These results might help elucidate the mechanism of fruiting differentiation and improve breeding efficiency.

## Introduction

*Pleurotus eryngii* is a white rotter of wood and the only species of its genus that grows on the living stems and roots of umbellifers [1]. *Pleurotus* sp. mushrooms have rapidly emerged as one of the most popular cultivated edible mushrooms [2]. In particular, *P. eryngii*, the king oyster mushroom, has recently become a major cultivated mushroom in Korea, China, and Japan because of its good taste, flavor, and nutritional factors. As the consumption of *P. eryngii* increases, new strains need to be bred with improved traits. However, mating incompatibility occasionally limits the efficiency of breeding programs in which crossing within loci-sharing strains or backcrossing strategies are employed. When a specific strain is required to have a single useful trait such as disease resistance, backcrossing is considered to be the best method for introducing the trait into the recurrent parent. In a backcross, however, only 25% of all possible combinations are compatible in tetrapolar mushroom fungi. Therefore, a breeder cannot evaluate the traits of incompatible hybrids. Although the mating-type systems of several edible mushrooms have been examined [3], [4], most current knowledge has been derived from studies of model mushrooms such as *Schizophyllum commune* and *Coprinopsis cinerea* [5], [6]. Thus, the mechanisms controlling the mating system in *P. eryngii* require further examination to improve the efficiency of *P. eryngii* breeding programs and to address its basic biology.

*P. eryngii* uses tetrapolar heterothallism as a part of its reproductive process. In a tetrapolar system, genes in the *A* locus encode a homeodomain transcription factor, whereas the *B* locus encodes both peptide pheromones and receptors (for review, see [7]). The structure of the *B* locus differs in different species. For example, in *Ustilago maydis*, a single locus contains one receptor and one pheromone, whereas *S. commune* possesses two functionally independent sub-loci containing one receptor and several pheromones [6]. In *C. cinerea*, a common structure is three sub-groups, with one receptor and multiple pheromones in each sub-group [5], [8], [9]. However, recent genome sequence reports have shown that the *matB* locus is much more complex than previously realized [4], [10], [11]. Some receptors referred to as receptor-like were found at loci distinct from the known mating loci, and until now, whether the receptor-like gene(s) had a role in the mating process was unclear.

Previously, sixteen alleles at locus *A* and 15 alleles at locus *B* were identified from the 12 strains of *P. eryngii*, and the *B3*-specific SCAR primers discriminating the *B3* locus were developed based on the RAPD (random amplified polymorphic DNA)-derived sequence, which was specifically amplified from *B3* locus-harboring strains [12]. In this study, we isolated the mating-type *B3* locus of *P. eryngii* using a PCR-based library screening method with a specific marker. A physical gene map of the region controlling mating type with both flanking regions is presented here. The expression patterns of the pheromone and receptor genes were analyzed in both monokaryotic and dikaryotic mycelia. Functional analyses of the pheromone and receptor genes were performed by *Agrobacterium*-based transformation. To the best of our knowledge, this study is the first functional analysis of pheromone and receptor genes in edible mushrooms.

## Materials and Methods

### Fungal strains and growth conditions

Two monokaryon strains, P5 and P6 (Table 1), were obtained from the dikaryotic mushroom KNR2312 using de-dikaryotization through protoplast homogenization [12] and has been deposited at Korean Culture Center of Microorganisms as KCCM90103 (P5) and KCCM90104 (P6). Cultured mycelia were obtained by inoculating 3–4 pieces of 1×1 cm^2^ MCM (mushroom complete medium: 0.2% peptone, 0.2% yeast extract, 2.0% glucose, 0.05% MgSO_4_·7H_2_O, 0.05% K_2_HPO_4_, and 0.046% KH_2_PO_4_) agar with mycelia in MCM broth, followed by incubation for 14 days at 25°C and 120 rpm. After incubation, the mycelia were filtered with sterile Whatman filter paper No. 41 and lyophilized before grinding with a sterile mortar. For long-term storage, strains were cultivated on slant medium of MCM, filled with sterile mineral oil (Sigma, St. Louis, MO, USA), and placed at 4°C until further use.

**Table 1:** *Pleurotus eryngii* strains used in this study and their mating interactions.

Species	Description	Note (reference)^a^
KNR2312	dikaryon, P5 (*A4B3*)+P6 (*A3B4*)	
P5	monokaryon, *A3B4*	protoclone [12]
P6	monokaryon, *A4B3*	protoclone [12]
KNR2312-4	monokaryon, *A3B3*	meiotic spore [12]
KNR2312-6	monokaryon, *A3B4*	meiotic spore [12]
KNR2312-29	monokaryon, *A4B3*	meiotic spore [12]
KNR2312-2	monokaryon, *A4B4*	meiotic spore [12]
P5phb31	monokaryon, *A3B4::PEphb3.1*	transgenic, this study
P5phb33	monokaryon, *A3B4::PEphb3.*3	transgenic, this study
P5STE331	monokaryon, *A3B4::PESTE3.3.1*	transgenic, this study
P5phb31×KNR2312-2	dikaryon, positive control	this study
P5phb33×KNR2312-2	dikaryon transformation	this study
P5STE331×KNR2312-2	dikaryon transformation	this study
P5×KNR2312-2	negative control	this study

a Protoclones were prepared from the homogenization of protoplast [12]; meiotic spores were derived from meiotic recombination.

### Fosmid library construction and screening

High-molecular-weight P6 DNA was purified using the *Neurospora* SDS buffer method as described previously [13], with minor modifications. First, 1 g of lyophilized mycelium powder was added slowly to 10 mL of *Neurospora* SDS buffer (0.15 M NaCl, 0.1 M EDTA, and 2% SDS, pH 9.5) in a 125-mL flask. Second, 0.5 mL of 2 mg/mL proteinase K (Fermentas, Waltham, MA, USA) solution was added, followed by incubation at 37°C for up to 24 h with gentle agitation; 10 mL of sterile water was added to the slurry, and the cellular debris was removed by centrifugation at 15,344× *g*. The supernatant was extracted 3–5 times with phenol saturated with Tris-HCl (pH 8.0) and once with water-saturated chloroform. DNA was precipitated by adding 2.5 volumes of absolute ethanol and removed with a sterile glass rod into a 1.5-mL centrifuge tube. After briefly air-drying, 1 mL of sterile TE buffer (10 mM Tris-EDTA, pH 8.0) containing 30 µg/mL RNase A (Qiagen, Hilden, Germany) was added. After incubation at 37°C for several hours with occasional mixing by inversion, the genomic DNA (gDNA) solution was stored at 5°C until use. The gDNA was used to construct a library using the CopyControl Fosmid library production kit (Epicentre Biotechnologies, Madison, WI, USA) according to the manufacturer's instructions.

Screening of clones harboring the mating-type region from the fosmid library was conducted using direct PCR; 25 clones were pooled in LB+chloramphenicol (12.5 µg/mL) using sterile toothpicks, followed by incubation for 24 h at 37°C and 120 rpm. Plasmid DNA was extracted with the GeneAll EXPREP plasmid SV MINI Kit (GeneAll Biotechnology, Seoul, Korea) according to the manufacturer's protocol. The *B3*-specific SCAR primer set (Table S1) was used to specifically amplify strains harboring the *B3* locus. The thermal cycling reaction was conducted in 20-µL volumes using GoTaq (Promega, Madison, WI, USA) in a Dyad thermal cycler (Bio-Rad, Hercules, CA, USA) with the following parameters: initial denaturation at 94°C for 4 min, followed by 35 cycles of denaturation at 94°C for 1 min, annealing at 65°C for 1 min, and extension at 72°C for 1 min 30 s. Two clones carrying the *B3* locus were isolated by further PCR with individual clones displaying the SCAR marker band.

### Fosmid sequencing and data analysis

To isolate fosmid DNA, a QIAprep 96 Turbo Miniprep kit (Qiagen) was used. Two fosmids containing mating type-specific regions were sequenced by the shotgun method. Purified DNA was sheared into random fragments. After end repair and size fractionation, a DNA fragment was ligated into pUC118/*Hin*cII (Takara, Shiga, Japan) and then sequenced using the Big-Dye cycle sequencing kit and an ABI3700 or 3730xl DNA analyzer (Applied Biosystems, Foster City, CA, USA). Base calling, contig assembly, and editing were conducted using the phred/phrap/consed package (University of Washington, USA).

Genes in the mating type-specific region were identified using DNAMAN (Lynnon Biosoft, Quebec, Canada) and FgeneSH (Softberry, http://linux1.softberry.com/berry.phtml) with a *C. cinerea* model; the BLASTX function of the NCBI database was used to identify open reading frames (ORFs) and genes. ORFs with small peptide sequences (10–100 amino acids) were manually curated to identify the CaaX motif in the C-terminus and were compared with known sequences in the NCBI database using BLAST to identify small genes such as those encoding pheromones. Additionally, the characterization and identification of putative mating-type genes were manually curated using the NCBI BLASTX and Protein BLAST databases (USA). Transmembrane motif analysis of receptor genes from *P. eryngii* and other receptor sequences retrieved from GenBank were predicted using PHOBIOUS (http://phobius.sbc.su.se/), HMMTOP (http://www.enzim.hu/hmmtop/), and TMHMM (http://www.cbs.dtu.dk/services/TMHMM/) software. Phylogenetic analysis was performed according to a previous report [4]. The receptor sequences of *S. commune*, *P. djamor*, *C. cinerea*, and *U. maydis*, available in the NCBI GenBank database, were used with previously reported accession numbers [14]. The receptor sequence of *Laccaria bicolor* was obtained from a genome site at the Joint Genome Institute (JGI: http://genome.jgi-psf.org/Lacbi2/Lacbi2.home.html) according to an accession number listed in a previous report [15]. The *U. maydis* PRA2 was used as an out-group. Protein sequences were aligned using ClustalW 1.64, and maximum likelihood analyses were conducted to estimate phylogenetic relationships using RAxML 7.04. Branch support was inferred from 1000 repetitions of nonparametric bootstrapping.

### RNA extraction and cDNA synthesis

Total RNA from KNR2312 (dikaryon) and P6 (monokaryon) of *P. eryngii* were extracted from lyophilized mycelia using modified protocols for TRIzol (Invitrogen, Carlsbad, CA, USA) and the RNeasy Mini Kit (Qiagen). After extracting total RNA, the RNase-Free DNase set (Qiagen) was used to eliminate gDNA contamination. Both sets of RNA were evaluated for quantity and quality using a NanoDrop Spectrophotometer ND-1000 (NanoDrop Technologies, Wilmington, DE, USA). The remaining samples were flash-frozen in liquid nitrogen and stored at −70°C until use. Then, the isolated RNA was transcribed into cDNA using a reverse transcription system with oligo-dT primer (Promega). The converted cDNA was used for both sequence verification and gene expression analysis. Four putative pheromone precursor genes and 4 receptor genes were amplified from the P6 cDNA using specific primers (deposited with the cDNA sequences in GenBank). PCR products purified from the gel using the QIAquick PCR Purification Kit (Qiagen) were transformed into *Escherichia coli* plasmids using the TOPO cloning kit for sequencing (Invitrogen) and the pGEM-T Easy Vector (Promega). Plasmids in transformed *E. coli* were isolated using Hybrid-Q (GeneAll Biotechnology) and prepared for sequencing by the GenoTech Company (GenoTech Corp., Daejeon, Korea). After qualifying the cDNA sequences, the four pheromone and four receptor gene sequences, along with their primers for amplification, were deposited in GenBank under the following accession numbers: KC879316 for *PESTE3.3.1*, KC879317 for *PESTE3.3.2*, KC879318 for *PESTE3.3.3*, KC879319 for *PESTE3.3.4*, KC879320 for *PEphb3.1*, KC879321 for *PEphb3.2*, KC879322 for *PEphb*3.3 and KC879323 for *PEphb3.4*.

### Analysis of gene expression by real-time qRT-PCR

Real-time qRT-PCR (CFX96, Bio-Rad) was performed using KNR2312 (dikaryon) and P6 (monokaryon) to quantify the expression of the 4 receptor genes and the 4 pheromone genes. The primer pairs used for real-time qRT-PCR amplification of the internal fragments of these genes are listed in table S2. Synthesized cDNA (100 ng/ul) was used as a template for real-time qRT-PCR. Real-time qRT-PCR was run as a duplex, in which one duplex partner was used as the internal standard gene (18S rRNA of *P. eryngii*), with the accession number FJ572254. The other duplex partner was one of the 4 pheromone genes or 4 receptor genes. The Rotor-Gene Q (QIAGEN, Hilden, Germany) was used for the reaction as follows: an initial step at 95°C for 5 min, followed by 39 cycles at 95°C for 5 s and 62°C for 10 s. One microlitre of cDNA was amplified in a 25.0 µl reaction using Rotor-Gene SYBR Green PCR kit master mix (Qiagen) with each primer at a final concentration of 500 nmol/l. The Ct value of each gene was normalized for differences in the amount of total cDNA in the reaction using the 18S rRNA of *P. eryngii* as an internal standard control. The normalized values for the pheromones and receptors were then calibrated to the value for the monokaryon (P6), which was assigned a value of 1, by the standard curve method using the Rotor-Gene software (QIAGEN). All assays were performed in triplicate. The statistical analysis of the gene expression was performed by one-way analysis of variance (ANOVA) and Tukey's test (alpha = 0.05) using the SAS program (SAS 9.1, SAS Institute Inc., Cary, NC, USA).

### Agrobacterium-mediated transformation

Genomic fragments containing a 1-kb upstream fragment for the promoter and a 0.7-kb downstream fragment for the terminator of two pheromone genes (*PEphb3.1* and *PEphb3.*3) and one receptor gene (*PESTE3.3.1*) were ligated into the *Sal* I site of the pBGgHg binary vector carrying the hygromycin B resistance gene (*hph*) [16]. Although this vector has the *Agaricus bisporus* gpdII promoter for expression, we adapted each gene's own promoter and terminator. Recombinant pPEphb3.1, pPEphb3.3 and pPESTE3.3.1 were introduced into *Agrobacterium tumefaciens* AGL-1 and transformed into *P. eryngii* P5 as described previously [17], with minor modifications. *Agrobacterium* induction medium (IM) culture and mycelium (harvested from colonies grown on MCM and homogenized for 30 s) culture (100 µL each) were co-cultivated, followed by treatment with sonication for 3 min and vacuum for 30 min, and then inoculated overnight at 25°C in darkness.

Putative transgenic lines showing hygromycin resistance were confirmed by PCR using specific primers (Table S1) as described previously [17]. The SSR45 primer set showing co-dominant polymorphism on KNR2312, P5 and P6 [18] was used to confirm the monokaryon or dikaryon (hybrid) P5 transformants.

### Test crossing and fruiting

To verify the biologically active transformed genes, transformants were crossed with monokaryotic tester strains harboring 4 different mating types (Table 1). Crosses between monokaryotic cultures were performed by placing actively growing mycelial plugs approximately 1 cm apart in the center of a Petri dish of MCM [12]. The mycelia were screened for clamp connections under 40× magnification at the growing boundary lines of either side of the interacting strains. Fruiting tests for all crosses were performed as described by Ryu *et al.* [12].

## Results

### Isolation and sequence analysis of the mating type-specific region

**Figure 1:**
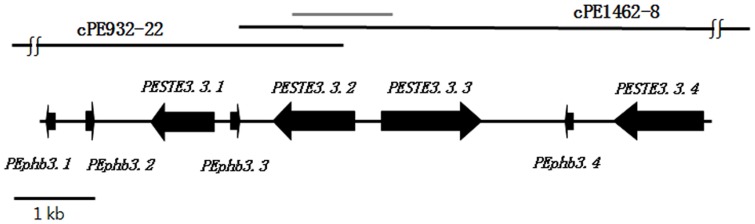
Physical map of the *B3* locus. The gDNA sequence of the *B3* locus is covered by two overlapping fosmids, cPE932-22 and cPE1462-8. The genes identified in the sequences are shown as a horizontal wide arrow. The arrows indicate the direction of transcription. The gray bar on the map represents the sequence amplified by the *B3* locus-specific primers (13-2_2100_ R, F). *PESTE3.3.1* stands for *Pleurotus eryngii* STE3 at the *B* 3 locus and 1 ^st^ (serial number) gene, *PEphb3.1* stands for *Pleurotus eryngii* pheromone at the *B3* locus and 1 ^st^ (serial number) gene.

The *B3* mating-type locus was isolated using PCR amplification with the *B3*-specific SCAR primer sets from a genomic library (more than 8,850 fosmid clones) constructed from KNR2312. DNA inserts of *P. eryngii* in cPE932-22 and cPE1462-8 were subcloned and sequenced using the shotgun method. Only the subclones containing 2–4 kb of DNA were sequenced in the forward and reverse directions using universal sequencing primers. The sequence data were edited, aligned, and merged using software. The sequence sizes in cPE932-22 and cPE1462-8 were 40.4 kb and 32.7 kb, respectively, and the total size of the sequences in the two clones was approximately 60 kb, excluding overlapping sequences. The SCAR marker [12] is located at the center of the locus (Figure 1), where the sequences from the two clones overlapped; thus, the two fosmid clones likely covered the complete *B3* locus.

### Identification of the pheromone and pheromone receptor genes on the *B3* locus

ORFs and their putative functions were identified in the entire 60-kb *B3* locus using BLASTX and FgeneSH (Softberry). A physical map of the *B3* locus is shown in Figure 1. The four pheromone and four receptor genes in the *B3* locus of KNR2312 spanned less than 12 kb. To verify the existence of subloci in KNR2312, flanking sequences were subjected to TBLASTX and contained additional open reading frames, but none of the encoded proteins showed sequence similarity to known pheromone or receptor genes (Table 2). However, we did not exclude th possibility that there are *B3* subloci in KNR232 even though there was no non-parental *B* locus in the 98 meiotic monokaryons from KNR2312 (12).

**Table 2:** Gene homologs identified in the *P. eryngii B3* mating-type locus and their flanking regions.

Gene	*E* value^a^	Position	Homolog	Possible function
*rluF*	4.3	245–2109	ribosomal large subunit pseudouridine synthase F [*Flavobacteria bacterium* BBFL7] (ZP_01201616.1)	responsible for the synthesis of pseudouridine from uracil-2604 in 23S ribosomal RNA
*Pol*	2e^−62^	3536–4072	DNA/RNA polymerase, partial [*Trametes versicolor* FP-101664 SS1] (EIW61623.1)	DNA repair
*Pol*	3e^−99^	5603–6601	reverse transcriptase-RNase H-integrase [*Tricholoma matsutake*] (BAA78625.1)	DNA integration and RNA-dependent DNA replication
	1.7	9893–10651	conserved hypothetical protein [*Trichophyton verrucosum* HKI 0517] (XP_003018684.1)	unknown
*CYP*	2e^−82^	13695–15784	cytochrome P450 [*Phanerochaete chrysosporium*] (BAL05081.1)	catalysis of the oxidation of organic substances
*omt*	2e^−85^	16301–23699	O-methyltransferase [*Punctularia strigosozonata* HHB-11173 SS5] (EIN06883.1)	catalysis of the transfer of a methyl group to the oxygen atom of an acceptor molecule
*rnt*	0.03	24383–25349	ribonuclease T [*Bermanella marisrubri*] (ZP_01305716.1)	catalysis of the endonucleolytic cleavage into nucleoside 3′-phosphates and 3′-phosphooligonucleotides ending in Gp with 2′,3′-cyclic phosphate intermediates.
*PEphb3.1*	6.6	26325–26510	fungal mating-type pheromone [*Coprinopsis cinerea* okayama7#130] (XP_002910434.1)	mating type-specific peptide pheromone
*PEphb3.2*	1.7	27056–27232	B mating type pheromone precursor [*Ceriporiopsis subvermispora* B] (EMD35227.1)	mating type-specific peptide pheromone
*PESTE3.3.1*	6e^−151^	28186–29384	putative pheromone receptor [*Flammulina velutipes*] (AGG68705.1)	mating type-specific G-protein-coupled receptor
*PEphb3.3*	9.7	29629–29877	fungal mating-type pheromone [*Coprinopsis cinerea* okayama7#130] (XP_00183497.1)	mating type-specific peptide pheromone
*PESTE3.3.2*	4e^−151^	30416–31911	putative pheromone receptor [*Flammulina velutipes*] (AGG68705.1)	mating type-specific G-protein-coupled receptor
*PESTE3.3.3*	5e^−109^	32405–34232	hypothetical fungal pheromone GPCR, STE3-type [*Postia placenta* Mad-698-R] (XP_002471247.1)	mating type-specific G-protein-coupled receptor
*PEphb3.4*	0.46	35693–35869	lipopeptide mating pheromone precursor bap3-1 [*Schizophyllum commune* H4-8] (XP_003028251.1)	mating type-specific peptide pheromone
*PESTE3.3.4*	1e-^129^	36579–38224	pheromone receptor Rcb2 B43 [*Coprinopsis cinerea* okayama7#130] (XP_002910431.1)	mating type-specific G-protein-coupled receptor
	1e^−74^	38948–41569	hypothetical protein SERLA73DRAFT_78193 [*Serpula lacrymans* var. lacrymans S7.3] (EGN93817.1)	unknown
*ubiA*	3 e^−30^	43157–43972	UbiA prenyltransferase [*Saprospira grandis* str. Lewin](YP_005323559.1)	integral to membrane
	9 e^−20^	44187–45557	predicted protein [*Laccaria bicolor* S238N-H82] (XP_001881105.1)	unknown
*sgnH*	2 e^−170^	46375–48010	SGNH hydrolase [*Trametes versicolor* FP-101664 SS1](EIW56391.1)	lipid metabolic process
	0	48339–51782	hypothetical protein SERLA73DRAFT_172732 [*Serpula lacrymans* var. lacrymans S7.3] (EGN92622.1)	unknown
	0	53063–55751	hypothetical protein SERLA73DRAFT_190801 [*Serpula lacrymans* var. lacrymans S7.3] (EGN92621.1)	unknown
*Pol*	0	57385–59974	reverse transcriptase-RNase H-integrase [*Tricholoma matsutake*] (BAA78625.1)	DNA integration and RNA-dependent DNA replication

a The expected value (*E*) indicates the number of hits one can “expect” to see by chance in GenBank similarity searches. Under “Homolog”, the species form with the lowest *E*-value hit or the value closest to zero is given in brackets, followed by the GenBank accession number in parentheses.

The *CLA4*, AKOR1, and AKOR2 genes, which are located next to or near the *B* mating locus in *Pleurotus djamor* [14], were not identified at −26.3 to +21.7 kb relative to the *B3* region (Table 2). Instead, cytochrome P450 (*CYP*) and reverse transcriptase-RNase H-integrase (*pol*) was identified upstream of the *B3* region.

All receptors contain a 7-transmembrane (TM) motif with varying amino acid lengths: 328 for PESTE3.3.1, 420 for PESTE3.3.2, 558 for PESTE3.3.3, and 470 amino acids for PESTE3.3.4. Alignment analysis revealed that the identities between PESTE3.3.1 and PESTE3.3.2 were 64.8% (full length) and 83.4% (compared with the truncated PESTE3.3.2 [326 amino acids], which did not include the non-corresponding C-terminal sequence of PESTE3.3.1). Comparison analysis showed that the C-terminal cytoplasmic domains varied in length across the genus, species, and even within a locus. PESTE3.3.1 has only 34 amino acid residues in its C-terminal cytoplasmic domain (Table S3). The C-terminal cytoplasmic domain of *S. commune* was relatively long (326–344 amino acids), whereas those of *U. maydis* and *Lentinus edodes* were 54 and 26–35 amino acids long, respectively.

**Figure 2:**
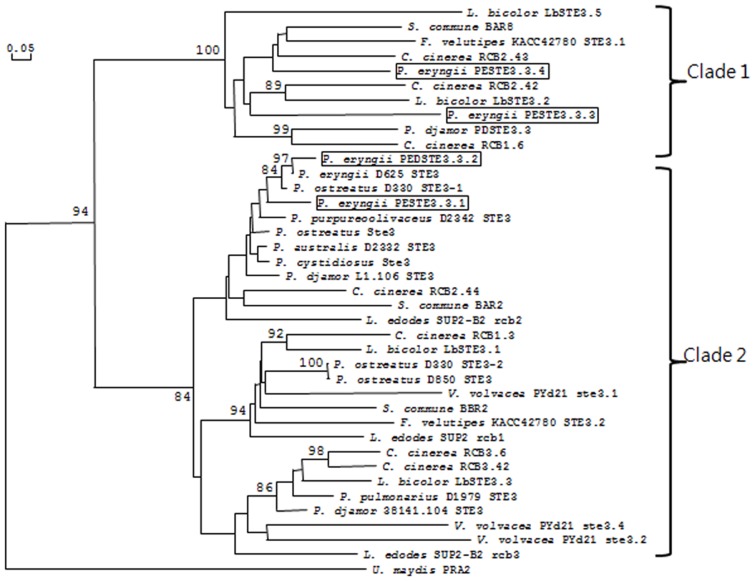
Phylogenetic relationships of the fungal pheromone receptor protein sequences. CC: *C. cinerea*; SC: *S. commune*. rcb1, rcb2, and rcb3 from *C. cinerea*; Bα and Bβ from *S. commune*; PDSTE3.3 from *P. djamor*; PESTE3-like from *P. eryngii*; and LBSTE3-like from *L. bicolor*. The GenBank accession numbers of the receptors analyzed here are shown in Table 2 and were previously reported [14], except the following; *L. edodes* SUP2 B2 rcb2: AER51012.1; *V. volvacea* ste3.1: JX982139.1; *V. volvacea* ste3.2: JX982140.1; *V. volvacea* ste3.3: JX982141.1; *V. volvacea* ste3.4: JX982142.1; *F. velutipes* W23 ste3.2: KC208605.1; *F. velutipes* W23 ste3.4: KC208611.1; *F. velutipes* KACC42780 ste3.1: HQ630590.1; and *F. velutipes* KACC42780 ste3.2: HQ630591.1.

Using maximum likelihood algorithms, the molecular phylogenetic analysis of receptors from *P. eryngii* and several mushroom fungi, which are available in GenBank (http://www.ncbi.nlm.nih.gov/genbank/), showed that two distinct clades were grouped with basal STE-like proteins such as *U. maydis* PRA2 (Figure 2). Clade 2 was roughly sub-divided into 3 groups, with not all having strong bootstrap support (>80). PESTE3.3.1 and PESTE3.3.2 are part of clade 2, which were similar to *P. ostreatus* D330 STE3-1 and *P. eryngii* D625 STE3, whereas PESTE3.3.3 belongs to clade 1 with *L. bicolor* LbSTE3.2 and *C. cinerea* RCB2.42. PESTE3.3.4 belonged with the receptors of clade 1 along with the orthologous *C. cinerea* RCB2.43 and *S. commune* BAR8. Sub-clusters containing *P. eryngii* receptors possessed a known mating-type gene, indicating that the receptor genes from *P. eryngii* likely evolved from the common ancestor of the known mating genes. In particular, *L. edodes* receptors were found in a basal position in every subgroup of clade, suggesting that the sequences of *L. edodes* receptors are most likely closer to the ancestral gene than the other receptor pheromone sequences in clade 2.

**Figure 3:**
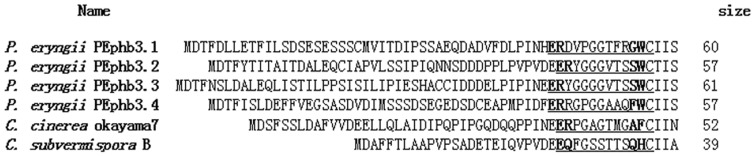
Alignment of the pheromone precursor amino acid sequences. The processed pheromone sequences are underlined, and the putative C-terminal doublets (ER) are shown. The GenBank accession number of the *C. cinerea* okayama 7 pheromone is XP_001834397.1, and that of *Ceriporiopsis subvermispora* B is EMD35227.1.

Fungal pheromone genes are sufficiently different to not hybridize with other genes, but they share consensus sequences in the C-terminal end of the protein precursors, the CaaX motif in *C. cinerea* and the CXXX motif (CVCH, CVRG, CVVA) in *S. commune* [9]. CaaX is conserved for post-translational modification in most pheromones; therefore, it can be used to identify the clade of the pheromone. We translated all possible peptides and evaluated the CaaX sequence at the C-terminal end. In the *P. eryngii B3* locus, four pheromone precursors were identified using DNAMAN (Lynnon Biosoft, Cambridge, UK) and BLASTP. A BLASTP search showed that each pheromone precursor has a similar precursor, including *C. cinerea* phb3.2 in *B43* (XP_002910434.1) for PEphb1.1, the *Ceriporiopsis subvermispora* B mating-type pheromone precursor (EMD35227.1) for PEphb3.2, *C. cinerea* fungal mating-type pheromone (XP_001834397.1) for PEphb3.3, and phb3 in the *B5* locus in *C. cinerea* (AAQ96360.1) for PEphb3.4. The 4 pheromone precursors contain 57–61 amino acids and have a cysteine residue at the 4th site from the C-terminus. During post-translational modification, 3 amino acid residues at the C-terminus are removed, and a 4th cysteine residue is farnesylated. Mature pheromones were 9–15 amino acids in length [8], [21] in the *B3* locus; the mature pheromone size was assumed to be 12 to 13 amino acids based on the consensus sequences for the excision site (Figure 3). Additional consensus sequences were found at the ER or TR at the 9–15th sites from the C-terminus and at a proline at the −4 and aspartate/asparagine at −2 relative to the cutting site. These conserved sites were recognized by processing molecules [9]. Sequences are typically more diverse across a genus and species than within a species. The cDNA sequences of the pheromone and receptor genes were deposited in GenBank with the primers used for their amplification.

### Gene expression of the pheromone receptor and pheromone genes in monokaryons and dikaryons

**Figure 4:**
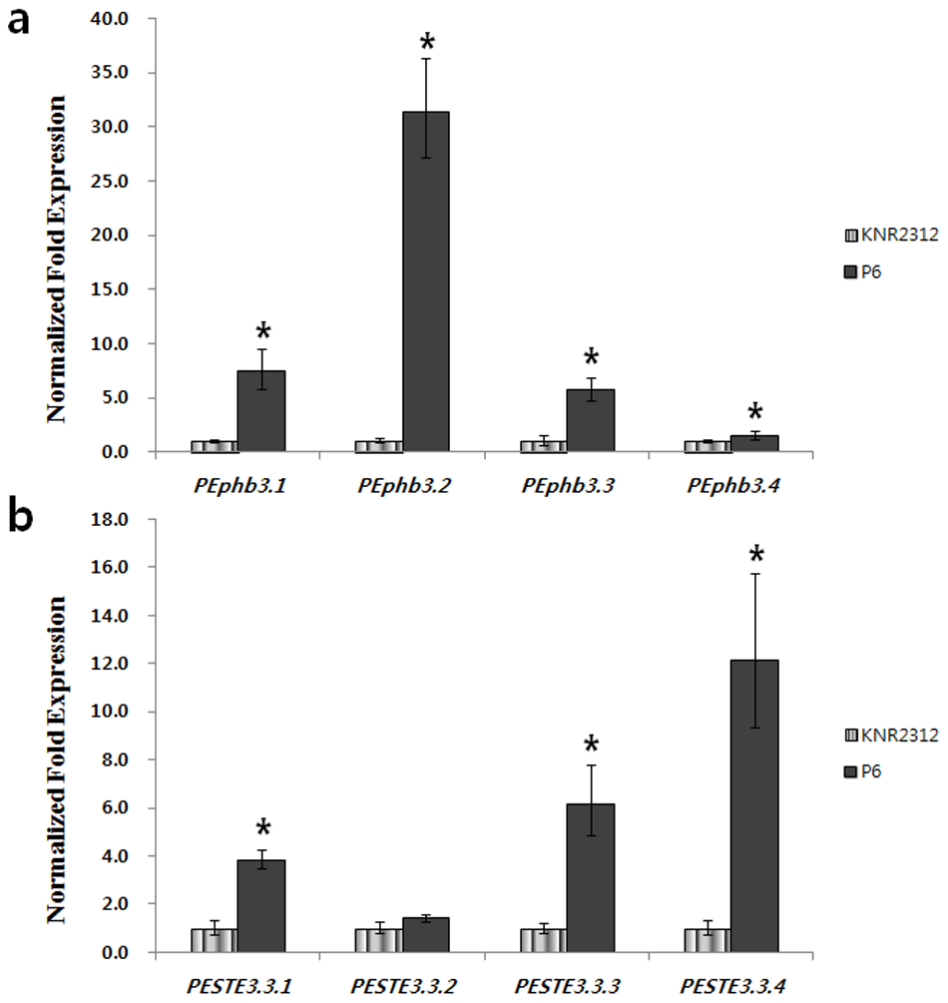
Relative expression of *P. eryngii* KNR2312 receptors (a) and pheromones (b) in the monokaryon (P6) and dikaryon (KNR2312), as determined by real-time qRT-PCR. Gene expression was normalized to 18S rRNA expression and calibrated to the value for the monokaryon (P6), which was assigned a value of 1, by the standard curve method (Bio-Rad). All assays were performed in triplicate. The error bars show the standard deviations for triplicate samples.

Real-time qRT-PCR was performed with KNR2312 (dikaryon) and P6 (monokaryon) to quantify the expression of the 4 pheromone precursor genes and the 4 receptor genes. Gene expression was normalized to 18S rRNA expression and calibrated to the assigned value of 1 by the standard curve method (Qiagen). The transcription level of the receptor genes was significantly higher (95% confidence interval) in the monokaryon (P6) (3.8–12.2 fold) than in the dikaryon (KNR2312), except for *PESTE3.3.2* (Figure 4a, Table S4). The expression patterns of the pheromone precursor genes were similar to those of the receptor genes, ranging from 1.5–31.4 fold (Figure 4b, Table S4). In particular, *PESTE3.3.4* and *PEphb3.2* showed the most significantly different expression pattern between monokaryotic and dikaryotic mycelia.

### Function of pheromones and pheromone receptors in the fruiting process

**Figure 5:**
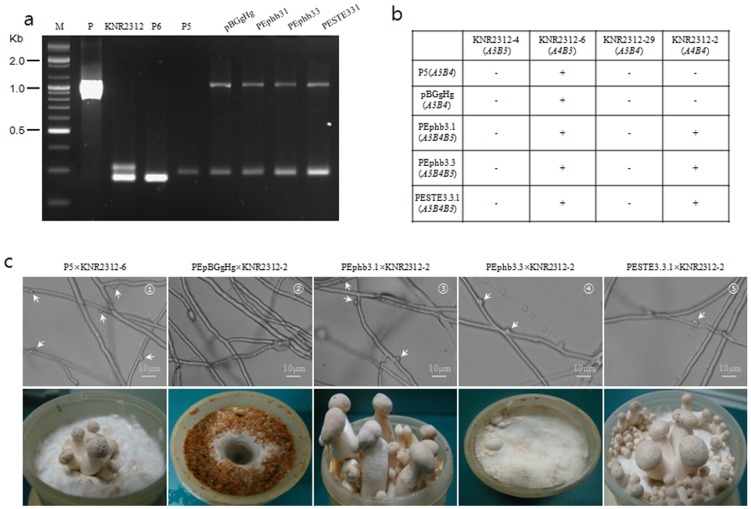
PCR band pattern for the confirmation of transformation, crossing table between the wild-type and transgenic monokaryons, and fruiting body (clamp connection) formation. a: Specific DNA fragments obtained in test-crossed mycelia using *hph* (Table S1). P: positive marker (pBGgHg), KNR2312: a dikaryon consisting of P5 and P6, P6: a monokaryon, P5: a monokaryon, pBGgHg: a transformant with the empty vector (pBGgHg), P5phb31: a P5 transformant with *PEphb3.1*, P5phb33: a P5 transformant with *PEphb3.3*, P5STE331: a P5 transformant with *PESTE3.3.1*, b: Wild-type tester strains (KNR2312-4: *A3B3*, KNR2312-6: *A3B4*, KNR2312-29: *A4B3*, and KNR2312-2: *A4B4*) were used for crosses with the transgenic monokaryons with the empty vector or with the pheromone or receptor genes, c: Clamp connections and fruiting bodies were observed in the positive control (wild type) (1) and dikaryons mated with transgenic monokaryons and the tester strain (3 and 5), but none were observed in the incompatible combination (2). In the case of 4, only primordia were shown. Labels above the panels indicate the cross combinations.

The transgenic monokaryon P5 was developed using two pheromone genes and one pheromone receptor gene by *Agrobacterium*-mediated transformation. Twelve transformants of P5phb31, 16 of P5phb33 and 3 of P5STE331 were screened using hygromycin selection medium and were confirmed by PCR with *hph*-specific primers. PCR with the SSR45 primer set produced one single band identical to that of P5, indicating the monokaryotic nature of the transformants (Figure 5a). Clamp connections were observed in dikaryon crosses between three transformants and KNR2312-2 (*A4B4*) (Figure 5b and 5c (3)–(5)), whereas crosses between transformants and monokaryons (*A3B3* or *A3B4*) could not develop clamp connections due to incompatible combinations (Figure 5b). The control combination of P5 and the tester strain (*A4B3*) possessed clamp connections (Figure 5c (1)), whereas the transformant containing the pBGgHg vector was unable to form clamp connections when crossed with the *A4B4* tester strain (Figure 5c (2)) due to incompatibility at the *A* locus. The positive control developed the clamp connections within a week, whereas the transformed monokaryons required 30–40 days after the two mycelia contacted. Moreover, the density of the clamp connections was 2.5 times higher in transformed monokaryons than in the wild-type strain (Figure 5c). Nonetheless, on the basis of our results, the transformed pheromone and receptor genes appear to play a role in the clamp connection process.

All combined dikaryons, including the positive and negative control strains, were fruited in sawdust medium. Only compatible combinations showed fruiting bodies similar to those of the positive strain (Figure 5c). The dikaryons P5phb31×KNR2312-2 and P5STE331×KNR2312-2 showed full fruiting bodies, whereas those of P5phb33×KNR2312-2 did not fully differentiate and had primordia without caps. All fruiting bodies were relatively small and delayed (more than 25 days) compared with KNR2312 (18 days, data not shown).

## Discussion

### Structure of the *B3* locus

Mating-type genes show extraordinarily diverse sequences; thus, using sequence-based methods to isolate whole mating-type genes is difficult. Although whole genome sequencing has been widespread and most of the genes of interest are available in online databases, the pheromone and receptor genes have distinct sequences in each strain. Alternatively, genomic subtraction and positional cloning using mitochondria intermediate peptidase (*MIP*), *CLA4*, putative multidrug transporter (*mfs1*), and *PAB1* have been employed to isolate the mating region [5], [8], . Additionally, marker genes are sometimes located too far from the *B* mating region to isolate the entire sequence of interest [14]. Recently, we identified mating-type alleles in the *A* and *B* loci and developed SCAR primers specific to the *B3* locus. Moreover, the SCAR marker, shown to be located in the middle of the *B3* locus, was amplified in *AxB3* monokaryons from in 100 monokaryons [12]. We isolated the *B3* mating region and identified a variety of genes, including mating-type genes, by sequence analysis using Fgenesh, BLASTX, and manual curation. The *B3* locus of *P. eryngii* possesses one more receptor (four receptors) than the typical *B* locus in *C. cinerea* (three receptors). In *S. commune*, the *B* locus is divided into two subloci, *Bα* and *Bβ* [19], [20], whereas in *C. cinerea*, only a single *B* locus is present. More than three pheromone genes at the *B* locus have been reported in *C. cinerea*, *Flammulina velutipes*, and *L. bicolor*. In most of these cases, unlike the *B3* locus of *P. eryngii*, extra receptors (other than the three functional receptors) that did not co-segregate with the mating type were located outside the locus and were considered to be impaired remnants of duplications [4], [15]. According to the phylogenetic analysis, *PESTE3.3.1* and *PESTE3.3.2* appear to be very closely related (Figure 2), and the sequence identity (64.8% between intact sequences and 83.4% between truncated and intact sequences) suggests that these two proteins were most likely duplicated later than any other receptors in the *B3* locus. The expression of the receptor and pheromone genes was supported by real-time qRT-PCR data. However, due to the short length of the C-terminal cytoplasmic residues, *PESTE3.3.1* seems likely to be a receptor-like gene. Whether such gene types play a role in the interaction between the receptor and downstream molecules remains unclear. Although P5-STE3.3.1×KNR2312-2 showed sparse clamp connections and late fruiting compared with the positive control (P5×KNR2312-6), it produced similar fruiting bodies to that of the positive control, indicating that a receptor with short C-terminal cytoplasmic residues could play a role in the fruiting process. In the case of *S. commune*, the *B*-dependent mating reaction was limited and produced an abnormal fruiting body after the shortening of the intracellular C-terminus [23]. In contrast, the truncation of the C-terminal intracellular region of the *S. commune* receptors has not been observed to affect signal transduction [24]–[26]. Furthermore, this short cytoplasmic region was found in not only *P. eryngii* but also other mushrooms such as *U. maydis* and *L. edodes* (Table S3). In addition to the C-terminal tail, multiple sites including the intracellular loop domains in 7-TM are thought to be involved in mating-type specificity [24], [25], [27]. Further analysis is required to determine how the length of the cytoplasmic domain of the receptor functions in the mating process. Novel pheromone-receptor interactions presumably evolved through gene duplications of the prototype STE3-like gene and the pheromone gene and subsequent mutation of the duplicated genes (for review see [28]). Because single- or double-amino acid substitutions in receptors or pheromones can significantly alter interaction specificity [29], PESTE3.3.1 could be a candidate for new mating specificity.

### Gene expression of the pheromone receptor and pheromone genes in monokaryons and dikaryons

Few studies have been conducted to analyze the expression of mating-type genes in mushroom fungi. Monokaryon and dikaryon fungal states show different expression levels of the *A* and *B* loci genes. Richardson *et al.* observed constitutive expression of the *A* locus genes to a similar extent in both monokaryons and dikaryons of *C. cinereus* [30]. In another study [23], a different expression pattern was observed for the receptor and pheromone genes of *S. commune*; at 12 h (mid-point of massive dikaryosis) after mating, both genes were highly expressed compared with the initial stage; at 72 h (end of massive dikaryosis), the pheromone genes were expressed at 2-fold higher levels than in the monokaryotic state, whereas the receptor genes were expressed at similar levels in the monokaryotic and dikaryotic states. The truncated pheromone was expressed at a lower level in the dikaryon. In the present study, comparative expression of the receptors and pheromone precursor genes revealed that most pheromone precursor genes were highly expressed in the monokaryons compared with the dikaryons (Figure 4), indicating that the fungal monokaryon status requires additional mate-specific pheromone and receptor genes for a monokaryon–monokaryon interaction to generate a dikaryon. The roles of pheromone perception in *Saccharomyces cerevisiae* have been described previously in mate attraction and the formation of shmoo cells that grow towards their mating partner [31]. Similarly, in *Neurospora crassa*, pheromones and receptors were involved in the chemotropic polarized growth of female-specific hyphae (trichogynes) toward male cells of the compatible mating type [32], [33]. A dikaryon does not require this attraction towards a compatible gamete. Before the mating process, sufficient amounts of the pheromones and receptors are required because the opposite mating type can only be met randomly.

### Function of pheromones and pheromone receptors in the fruiting process

Fungal transformation, especially in edible mushrooms, is not easily achieved, although some methods have been developed [34], [35]. We adapted *Agrobacterium*-mediated transformation, thus succeeding in the isolation of several transformants. The dikaryons from crosses between transformants (*A3B4::PEphb3.1*, *A3B4::PEphb3.3*, *A3B4::PESTE3.3.1*) and a monokaryotic tester strain (*A4B4*) demonstrated clamp connections, suggesting that the transgenic genes most likely produced compatible combinations with the tester strain. Although clamp connections were formed more slowly and sparsely in the transgenic dikaryons than in the positive control (Figure 5c), the transgenic dikaryons showed full fruiting bodies, except P5phb33×KNR2312-2, which indicated the requirement for more components of the normal process. This result is consistent with a previous report [23], in which malformed fruiting bodies and spores were observed in the transgenic dikaryons. The small and delayed fruiting bodies were likely caused by selfing depression.

## Supporting Information

Table S1 **Primers for the isolation of the** ***B3*** **locus and the confirmation of transformation and expression.** List of specific primer sets for the isolation of the *B3* locus from the gDNA library, the confirmation of transgenic mycelia and the amplification of pheromone and receptor gDNAs with their promoter and terminator sequences.
(DOCX)

Table S2 **Primers for real-time qRT-PCR (SYBR Green) in this study.** List of specific primer sets for real-time qRT-PCR to address the expression profiles of the pheromone and receptor genes in mono- and dikaryotic mycelia.
(DOCX)

Table S3 **A predicted transmembrane motif of the pheromone receptors.** The table lists the transmembrane motifs of fungal pheromone receptors from *P. eryngii B3* and other mushroom fungi. ^a^ All have GenBank accession numbers, except LbSTE3.2 from JGI (http://genome.jgi-psf.org/Lacbi2/Lacbi2.home.html), ^b^ A transmembrane motif was predicted using PHOBIOUS, HMMTOP, and TMHMM, as described in the methods. ^c^ *P. eryngii*, ^d^ *L. bicolor*, ^e^ *P. djamor*, ^f^ *S. commune*, ^g^ *U. maydis*, *^h^ C. cinerea*, ^i^ *L. edodes*.
(DOCX)

Table S4 **Real-time a RT-PCR raw data of ct value in monokaryons and dikaryons.**
(XLSX)
