# Supplementary material for: Identification and Functional Analysis of Pheromone and Receptor Genes in the B3 Mating Locus of Pleurotus eryngii
Source: PLoS One. 2014 Aug 18;9(8):e104693. doi: 10.1371/journal.pone.0104693 (PMC4136793; doi:10.1371/journal.pone.0104693)
Supplement: Table S1 — Primers for the isolation of the B3 locus and the confirmation of transformation and expression. List of specific primer sets for the isolation of the B3 locus from the gDNA library, the confirmation of transgenic mycelia and the amplification of pheromone and receptor gDNAs with their promoter and terminator sequences. (DOCX) [file pone.0104693.s001.docx]

Table S1. The list of primers for isolation of *B3* locus, confirmation of transformation and expression.

**Oligo Name Sequence (5’-3’) Description**

Phb3.1-ex-F: ACGCGTCGACACGTTACGGATATTTAAATAGTG *PEphb3.1* for expression

Phb3.1_ex-R: ACGCGTCGACACGGTGGCAACCTCTCA

Phb3.3-ex_F: GCCCACACACACAATGGACACTTT *PEphb3.*3 for expression

Phb3.3-ex_R: AAGCTCGTCGTCATCGATACAGCA

Pep 3-2 F: ACGCGTCGACTATATTCCATTGTTCGAAGC *PESTE3.3.1* for expression

Pep 3-2 R: ACGCGTCGACTTCACTATTTAAATATCCGTAACG

gpd F: GAAGAAGCTTTA AGAGGTCCG C confirmation of transformation

hph R: GGCGACCTCGTATTGGGAATC

13-2_2100_ F: CAAAGGTCCCCCCGATAGTGAT *B3* specific SCAR primer set

13-2_2100_ R: CAGAGGTCCCAGCTGGAAGTGT
