# Supplementary material for: Identification and Functional Analysis of Pheromone and Receptor Genes in the B3 Mating Locus of Pleurotus eryngii
Source: PLoS One. 2014 Aug 18;9(8):e104693. doi: 10.1371/journal.pone.0104693 (PMC4136793; doi:10.1371/journal.pone.0104693)
Supplement: Table S2 — Primers for real-time qRT-PCR (SYBR Green) in this study. List of specific primer sets for real-time qRT-PCR to address the expression profiles of the pheromone and receptor genes in mono- and dikaryotic mycelia. (DOCX) [file pone.0104693.s002.docx]

Table S2. The list of primers for Real-time q-PCR (SYBR Green) in this study.

**Oligo Name Sequence (5’-3’) Target gene**

PE 18s F: GGGCATGCCTGTTTGAGTGTCATT 18 rRNA gene of *P. eryngii*

PE 18s R: AGCCAGACTCTATTCATGCGTGCT

Pep 1-1 F: GGCGCGAGCAACAGC *PEphb3.1* of *P. eryngii*

Pep 1-1 R: CCCGAGGGCGAGG

Pep 1-2 F: CCTCCCCATCCACGC *PEphb3.2* of *P. eryngii*

Pep 1-2 R: TATGTTCGGATCTCGTTTCG

Pep 2-1 F: GCCCACACACACAATGGACACTTT *PEphb3.*3 of *P. eryngii*

Pep 2-1 R: AAGCTCGTCGTCATCGATACAGCA

Pep 3-2 F: AAGGCTTGGACCTCGCC *PEphb3.4* of *P. eryngii*

Pep 3-2 R: TCGTTTGGGGGGATATGTG

Pep R1 F: TGCCTGCGATCTCATGCTCACAA *PESTE3.3.1* of *P. eryngii*

Pep R1 R: TCGTGGCTTCGCAAGCAATCACA

Pep R2 F: AAAGCTTTCTGGGCGGTCGTGAA *PESTE3.3.2* of *P. eryngii*

Pep R2 R: TGGTTGCTTGGGTTGCACGAAA

Pep R3 F: CAACCCGTTTCTCAACGCACAGTT *PESTE3.3.3* of *P. eryngii*

Pep R3 R: ACGAATGGATAAACGTTGGGCTGC

Pep R4 F: TCATTGGATCTCAATTCGCGCTGC *PESTE3.3.4*of *P. eryngii*

Pep R4 R: TCGAAACGGTGACCTTGCACGATA
