# Supplementary material for: Identification and Functional Analysis of Pheromone and Receptor Genes in the B3 Mating Locus of Pleurotus eryngii
Source: PLoS One. 2014 Aug 18;9(8):e104693. doi: 10.1371/journal.pone.0104693 (PMC4136793; doi:10.1371/journal.pone.0104693)
Supplement: Table S3 — A predicted transmembrane motif of the pheromone receptors. The table lists the transmembrane motifs of fungal pheromone receptors from P. eryngii B3 and other mushroom fungi. a All have GenBank accession numbers, except LbSTE3.2 from JGI (http://genome.jgi-psf.org/Lacbi2/Lacbi2.home.html), b A transmembrane motif was predicted using PHOBIOUS, HMMTOP, and TMHMM, as described in the methods. c P. eryngii, d L. bicolor, e P. djamor, f S. commune, g U. maydis, h C. cinerea, i L. edodes. (DOCX) [file pone.0104693.s003.docx]

Table S3. A predicted transmembrane motif of the pheromone receptors.

| Pheromone receptor (accession no.)^a^ | Position of transmembrane motif in^b^ | | | | | | | Cytoplasmic residues (a.a) |
| --- | --- | --- | --- | --- | --- | --- | --- | --- |
|  | 1 | 2 | 3 | 4 | 5 | 6 | 7 |  |
| PESTE3.3.1^c^ | 6-24 | 36-58 | 64-89 | 113-134 | 163-184 | 205-229 | 272-291 | 292-326: 34 |
| PESTE3.3.2^c^ | 6-26 | 38-58 | 64-89 | 113-134 | 162-184 | 205-229 | 272-291 | 292-420: 128 |
| PESTE3.3.3^c^ | 6-22 | 34-53 | 73-92 | 113-133 | 153-182 | 203-228 | 267-286 | 287-557: 270 |
| PESTE3.3.4^c^ | 6-24 | 36-54 | 66-89 | 110-129 | 149-176 | 210-233 | 266-285 | 286-470: 184 |
| LbSTE3.1^d^ (XP001888609.1) | 12-29 | 41-61 | 73-93 | 114-137 | 161-187 | 208-231 | 278-295 | 296-335: 39 |
| LbSTE3.2^d^ | 6-23 | 30-53 | 73-90 | 111-131 | 156-181 | 202-227 | 266-285 | 286-396: 110 |
| LbSTE3.3^d^ (XP001888610.1) | 6-25 | 37- 55 | 75- 94 | 114-135 | 163-185 | 206-229 | 273-292 | 293-409: 116 |
| LbSTE3.5^d^ (XP001886578.1) | 6-24 | 31-54 | 66-90 | 110-130 | 150-174 | 200-220 | 262-280 | 281-315:34 |
| PDSTE3.3^e^ (AAS46748.1) | 15-32 | 39-61 | 70-94 | 119-136 | 163-186 | 215-238 | 270-294 | 294-560: 266 |
| BAR2^f^ (CAA62595.4) | 6-24 | 36-55 | 70-90 | 102-124 | 152-174 | 195-219 | 262-280 | 281-627: 346 |
| BAR8^f^ (AAR99618.1) | 6-22 | 29-52 | 72-89 | 110-130 | 150-174 | 205-225 | 267-285 | 286-553: 267 |
| BBR2^f^  (AAD35087.1) | 6-28 | 40-60 | 72-91 | 112-136 | 164-186 | 207-231 | 274-293 | 294-629:335 |
| PRA2^g^  ( P31303.1) | 17-36 | 43-66 | 81-100 | 123-144 | 167-190 | 229-248 | 275-299 | 292-346: 54 |
| RCB1.3^h^  (AAO17255.1) | 12-31 | 38-57 | 77-97 | 117-137 | 161-182 | 203-226 | 270-289 | 290-552: 262 |
| RCB1.6^h^ (CAA71964.1) | 6-23 | 35-55 | 67-90 | 111-130 | 150-177 | 206-226 | 260-285 | 286-558:272 |
| RCB2.42^h^ (AAF01419.1) | 6-24 | 36-58 | 70-89 | 109-130 | 150-180 | 201-226 | 265-284 | 285-389:104 |
| RCB2.43^h^ (AAQ96345.1) | 6-25 | 32-54 | 74-92 | 113-133 | 153-177 | 205-228 | 269-288 | 289-483: 194 |
| RCB2.44^h^ (AAQ96344.1) | 6-25 | 37-59 | 71-91 | 112-131 | 151-178 | 208-229 | 272-291 | 292-422:130 |
| RCB3.42^h^ (AAF01420.1) | 12-32 | 44-66 | 78-98 | 119-138 | 169-188 | 217-240 | 281-300 | 301-433:132 |
| RCB3.6^h^ (CAA71962.1) | 12-30 | 42-62 | 74-94 | 115-134 | 166-188 | 209-232 | 276-295 | 296-423:127 |
| rcb1-SUP2^i^ (AER51018.1) | 6-26 | 38-57 | 72-92 | 113-132 | 152-177 | 198-221 | 265-284 | 285-306:21 |
| rcb3-SUP2-B2^i^ ([AER51017.1](http://www.ncbi.nlm.nih.gov/protein/355000178)) | 12-32 | 44-64 | 76-96 | 117-139 | 168-190 | 211-234 | 278-296 | 297-332:35 |
